# Results of Treating Mild to Moderate Knee Osteoarthritis with Autologous Conditioned Adipose Tissue and Leukocyte-Poor Platelet-Rich Plasma

**DOI:** 10.3390/jpm13010047

**Published:** 2022-12-26

**Authors:** Vilim Molnar, Eduard Pavelić, Željko Jeleč, Petar Brlek, Vid Matišić, Igor Borić, Damir Hudetz, Eduard Rod, Dinko Vidović, Neven Starčević, Martin Čemerin, David C. Karli, Dragan Primorac

**Affiliations:** 1 St. Catherine Specialty Hospital, 10000 Zagreb, Croatia; 2 Faculty of Medicine, Josip Juraj Strossmayer University of Osijek, 31000 Osijek, Croatia; 3 Department of Nursing, University North, 42000 Varaždin, Croatia; 4 School of Medicine, University of Split, 21000 Split, Croatia; 5 Department of Health Studies, University of Split, 21000 Split, Croatia; 6 Department for Traumatology and Orthopaedics, University Hospital Dubrava, 10000 Zagreb, Croatia; 7 Clinic for Traumatology, University Hospital “Sisters of Mercy”, 10000 Zagreb, Croatia; 8 School of Dental Medicine, University of Zagreb, 10000 Zagreb, Croatia; 9 School of Medicine, University of Zagreb, 10000 Zagreb, Croatia; 10 The Steadman Clinic, Vail, CO 81657, USA; 11 School of Medicine, Faculty of Dental Medicine and Health, Josip Juraj Strossmayer University Osijek, 31000 Osijek, Croatia; 12 Medical School, University of Rijeka, 51000 Rijeka, Croatia; 13 Medical School, University of Mostar, 88000 Mostar, Bosnia and Herzegovina; 14 Eberly College of Science, Penn State University, 517 Thomas St., State College, PA 16803, USA; 15 The Henry C Lee College of Criminal Justice & Forensic Sciences, University of New Haven, West Haven, CT 06516, USA; 16 Medical School REGIOMED, 96450 Coburg, Germany

**Keywords:** knee osteoarthritis, mesenchymal stem cells, stromal vascular fraction, adipose tissue, platelet-rich plasma

## Abstract

Knee osteoarthritis (KOA) is one of the most common musculoskeletal disorders. Much progress has been made in regenerative medicine for the symptomatic treatment of KOA, including products containing stromal vascular fraction (SVF) and platelet-rich plasma (PRP). The aim of this study was to evaluate clinical and radiological findings after the application of autologous conditioned adipose tissue (ACA) and leukocyte-poor PRP (LP-PRP) in patients with mild to moderate KOA. A total of 16 patients (eight male and eight female) with changes related to KOA on the magnetic resonance imaging (MRI), but without severe osteophytosis, full-thickness cartilage loss, or subchondral bone involvement were included in this study. Patients received an intraarticular, ultrasound-guided injection of ACA and LP-PRP. Clinical scores, including a visual analog scale for pain (VAS), Knee Injury and Osteoarthritis Outcome Score (KOOS), and Western Ontario and McMaster Universities Osteoarthritis Index (WOMAC) were evaluated at baseline and at the three and six month follow-ups showing a statistically significant improvements at three and six months post-intervention. Furthermore, the delayed gadolinium-enhanced MRI of the cartilage (dGEMRIC) indices were evaluated at baseline and at the three and six month follow-ups showing no significant changes after treatment with ACA and LP-PRP, which were actually equal to the dGEMRIC indices measured in the control group (hyaluronic acid applied in contralateral knees without osteoarthritis). ACA with LP-PRP presents a viable minimally invasive therapeutic option for the clinical improvement of mild to moderate KOA. However, MFAT produced by different systems is likely to differ in cellular content, which can directly affect the paracrine effect (cytokine secretion) of mesenchymal stem cells and consequently the regeneration process.

## 1. Introduction

Osteoarthritis (OA) is one of the most common musculoskeletal pathologies with over 654 million patients worldwide, while this number is expected to rise due to an aging and a progressively obese world population [1]. It affects 21.7% of women and 11.9% of men over 40 years of age. The most predominantly affected joint in OA is the knee [2]. The high prevalence of this disease in older population groups, and the immense costs associated with this disease create a precarious situation for healthcare systems to find more affordable alternatives [3,4]. Due to the inefficiencies of the current nonoperative treatment of OA, there is a financial burden on both the patient and the healthcare system [4,5].

In recent years orthobiologic therapies offered great potential in the treatment of OA [6]. Options, such as the application of stromal vascular fraction (SVF) and microfragmented adipose tissue (MFAT) have shown encouraging results in the treatment of patients with knee osteoarthritis (KOA), including clinical and radiological improvements [7,8,9,10]. SVF is obtained through either mechanical or enzymatic degradation of lipoaspirate. Enzymatically, SVF can be obtained with the use of collagenase and centrifugation. Still the subject of discussion, mechanical isolation of SVF can be performed through a combination of centrifugation and intersyringe processing. Both methods have proven similar chondrogenic potentials in vivo [11]. If obtained correctly, SVF has shown excellent clinical results with a significant reduction in pain scores [12,13,14,15].

The alternative biological method that has been widely used in the past decade is the application of platelet-rich plasma (PRP). To be considered PRP, the platelet concentration must be 1,000,000 platelets/μL in a 5 mL volume of plasma [16]. However, there is significant heterogeneity in preparation systems of PRP, such as leukocyte-rich, leukocyte-poor PRP (LP-PRP), and others [17,18,19]. The plethora of growth factors present in PRP makes it an excellent therapeutic tool in the treatment of KOA [17,20,21,22]. Although PRP has shown great clinical results for patients with mild to moderate KOA, the current school of thought is to combine the two therapeutic methods to create a synergistic effect.

The aim of this study was to evaluate the effects of combination therapy with autologous conditioned adipose tissue containing SVF (ACA-SVF) and adjunct LP-PRP for mild to moderate KOA.

## 2. Materials and Methods

### 2.1. Study Design

This prospective, non-randomized, interventional, single-center, and open-label clinical study involved patients with primarily mild to moderate KOA who received a combination of ACA and LP-PRP. Mild to moderate KOA is defined as the presence of osteoarthritic knee changes but without diffuse full-thickness cartilage loss with underlying subchondral bone reactive changes in any joint surfaces (grade IV defects according to the International Cartilage Research Society (ICRS) based on the modified Outerbridge system). Clinical results were noted by filling out clinical questionnaires prior to intervention and 3 and 6 months after the intervention. All patients signed informed consent before being included in the study. The study was conducted in St. Catherine Specialty Hospital, Zabok, Croatia. We confirm that all methods were performed following the relevant guidelines and regulations. The study was approved by the St. Catherine’s Ethical Committee, authorization No: 21/3-1.

### 2.2. Participants

A total of 16 patients (8 male and 8 female) with KOA were included in this study. All the study participants were clinically examined by an orthopedic surgeon, and a magnetic resonance imaging (MRI) of the affected knee was performed along with standard knee X-rays. Unaffected knees, without KOA (contralateral knees of the same patients) were treated with hyaluronic acid (HA) (Hyalubrix^®^ 60, Fidia Farmaceutici S.P.A., Abano Terme, Italy) to compare the dGEMRIC indices with knees with KOA treated with ACA and LP-PRP.

Patient inclusion and exclusion criteria are described in Table 1.

### 2.3. Clinical Questionnaires

All patients were screened by an orthopedic examination following which they answered the orthopedic questionnaires related to KOA: the KOOS (Knee Injury and Osteoarthritis Outcome Score), the WOMAC (Western Ontario and McMaster Universities Osteoarthritis Index), and the pain level was assessed using a visual analog scale (VAS). For patients that were included in this study, clinical questionnaires were assessed at baseline and 3 and 6 months after the intervention. In the follow-up period, the patients were instructed to maintain normal daily activities.

### 2.4. Delayed Gadolinium-Enhanced Magnetic Resonance Imaging of Cartilage (dGEMRIC) Protocol

MR imaging was performed on a 1.5 T magnet (Avanto; Siemens, Erlangen, Germany) using a dedicated knee coil (Siemens, Erlangen, Germany). The severity of early OA in this study cohort was determined, according to the MRI, by an experienced musculoskeletal radiologist using the scoring system introduced by the International Cartilage Research Society (ICRS) based on the modified Outerbridge system.

After the completion of the clinical examination and the questionnaires, the patients were given intravenous contrast (gadolinium) to perform dGEMRIC.

Each subject received gadolinium diethylene triamine penta-acetic acid (Dotarem; Guerbet, Roissy CgG Cedex, Villepinte, France), 0.2 mmol/kg, administered with an injection time of less than 5 min through an IV infusion catheter placed in the antecubital vein with the patient in the supine position. The administered MRI contrast agent was the same for all patients because the MRI contrast agent was always applied under the same conditions: contrast agent temperature, magnetic field strength, and contrast agent concentration. The subjects waited 5 min after injection, then exercised by walking up and down the stairs and continued to walk on a flat surface for approximately 10 min to stimulate delivery of the contrast agent to the joint. Post-contrast imaging of the cartilage was performed 60 min after contrast administration. The dGEMRIC index was obtained by an experienced musculoskeletal radiologist using syngoMaplt software (Siemens, Erlangen, Germany). Seven different articular facets were analyzed, and the dGEMRIC index was calculated: the medial and lateral femoral condyle, femoral trochlea, medial and lateral tibial condyle, and both patellar facets, before the intra-articular application of stem cells and in any subsequent MRI examination at 3, and 6 months after the intra-articular application of ACA-SVF and LP-PRP. Regions of interest (ROIs), in which an average dGEMRIC index was calculated, were manually drawn to consistently cover the same weight-bearing part of each articular cartilage facet. Articular facets on which the dGEMRIC index was not measured were labeled as “-”.

### 2.5. Lipoaspiration and ACA Production

The patients were referred to the day surgery unit with an average admission of three hours. The surgical part of the procedure was performed in an operating theater. The patients were placed in a supine position; the abdominal skin was treated with antiseptic lotion Dermoguard^®^ (Antiseptica, Pulheim, Germany), rinsed with *Aqua pro injectione* solution (HZTM, Zagreb, Croatia), dried out, and disinfected with Skin-Des^®^ solution (Antiseptica, Pulheim, Germany). Injection of 2% lidocaine was administered to the incision site, after which a 2–3 mm incision was made. The minimally invasive surgical procedure included an infiltration step, in which a total of 250 mL of the saline solution was prepared with a 40 mL of a 2% lidocaine solution (Lidokain^®^, Belupo, Koprivnica, Croatia) and 1 mL epinephrine hydrochloride (1 mg/mL) (Suprarenin^®^, Sanofi-Aventis, Berlin, Germany) was injected into the abdominal subcutaneous adipose tissue. In the aspiration step, a standard lipoaspiration technique was performed, and the harvested fat was collected by a Carraway Harvester (2.1 mm × 15 cm) connected to a VacLock syringe (Arthrex, Munich, Germany) that was inserted through a small stab incision where up to 60 mL of adipose tissue was collected into the syringe by the vacuum created by the system. Steristrips (3M) were taped, and compression bandages were applied over the incisions to prevent hematoma formation.

The obtained lipoaspirate was divided into several (up to 4) separate syringes (Arthrex ACP^®^ Double-Syringe System (Arthrex, Munich, Germany)) and centrifuged for 4 min at 2500 rpm. Upon completion of centrifugation, 3 layers within the syringe were distinguished. The lowest layer, the aqueous fraction, was poured out, while the highest layer, the layer of broken adipocyte oil, was removed using the Arthrex ACP^®^ Double-Syringe System. The middle layer, a layer of autologous conditioned adipose tissue (ACA), was mixed with the same layers of the other syringes through a 1.4 mm wide transfer device at least 30 times to obtain a homogenized adipose tissue product (ACA Microfat). ACA Microfat was centrifuged again for 4 min at 2500 rpm. Again, the oil, which was in the upper layer, was separated and discarded, and the aqueous fraction poured out. The middle layer, consisting of ACA-SVF containing adipose-derived mesenchymal stem cells, was used as the final product for application to the patient’s knee joint. All the patients received 2 mL of ACA-SVF in combination with LP-PRP.

### 2.6. LP-PRP Protocol

A 90 mL sample of venous blood was taken from the patient to prepare LP-PRP using the Arthrex Angel System™. The settings were adjusted to increase the number of platelets to 5.57-fold while keeping the leukocyte and neutrophil levels at 0.78 and 0.53 in relation to normal venous blood. The final LP-PRP volume was set to 5 mL. In patients where less than 5 mL of LP-PRP was obtained, platelet-poor plasma (PPP) was added to the mixture so that the final volume was equal to 5 mL. 

### 2.7. Application of ACA + LP-PRP

Finally, the ACA (2 mL) was mixed with LP-PRP (5 mL) to form the final product. After disinfection of the puncture site, a 21-gauge needle was inserted into the synovial space of the knee joint guided by ultrasound. Synovial fluid was drawn from the knee, and a combination of ACA and LP-PRP was injected through the same needle used to aspirate the synovial fluid. Upon completion of the procedure, patients spent approximately 2 h in the hospital and were discharged home afterward.

### 2.8. Follow-Up Appointments

All patients arrived for a follow-up examination 3 and 6 months after the initial treatment. During these intervals, patients underwent magnetic resonance imaging (dGEMRIC) of the knee to determine the morphological and molecular state of the cartilage and its response to the combined ACA and LP-PRP therapy. Afterward, the patients answered the orthopedic questionnaires (VAS, KOOS, and WOMAC) to compare the clinical findings with the results before the initial treatment, thus assessing the clinical response to the ACA and LP-PRP therapy.

### 2.9. Statistical Analysis 

A statistical analysis of the obtained data was performed in the software package IBM SPSS Statistics 23.0 (SPSS, Chicago, IL, USA). Graphs were created in GraphPad Prism version 9.4.1. for Windows (GraphPad Software, San Diego, CA, USA). Descriptive statistical methods were used to describe the frequency of the investigated variables. The normality of the distribution of the variables was tested by the Kolmogorov–Smirnov test. We used Friedman’s test to compare three or more paired groups and the Wilcoxon matched pairs test to determine the differences between two paired groups (repeated measurements within the same group of subjects). 

## 3. Results

### 3.1. Visual Analog Scale (VAS)

**Figure 1:**
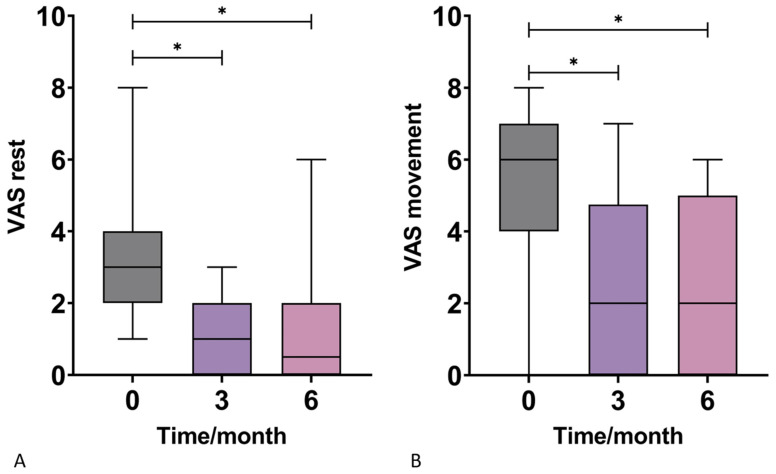
Box-plots show the distribution of visual analog scale (VAS) scores for pain in rest (**A**) and movement (**B**) at baseline and at 3 and 6 months after the application of autologous conditioned adipose tissue and leukocyte-poor platelet-rich plasma. Statistically significant VAS improvement in response to therapy was observed at 3 and 6 months at both rest and during movement. *—*p* < 0.05 (Wilcoxon test).

Patients were followed-up at three and six months for a score assessment during rest and movement. The results demonstrated no statistical difference in VAS scores between the two genders. However, there was a statistically significant VAS improvement in response to therapy at three and six months at both rest and during movement (Figure 1, Table 2). The mean scores for VAS in rest decreased from 3.00 to 1.00 at three months and then finally to 0.50 at six months. The mean scores for VAS in movement decreased from 6.00 to 2.00 at three and six months after the intervention.

### 3.2. Knee Injury and Osteoarthritis Outcome Score (KOOS)

**Figure 2:**
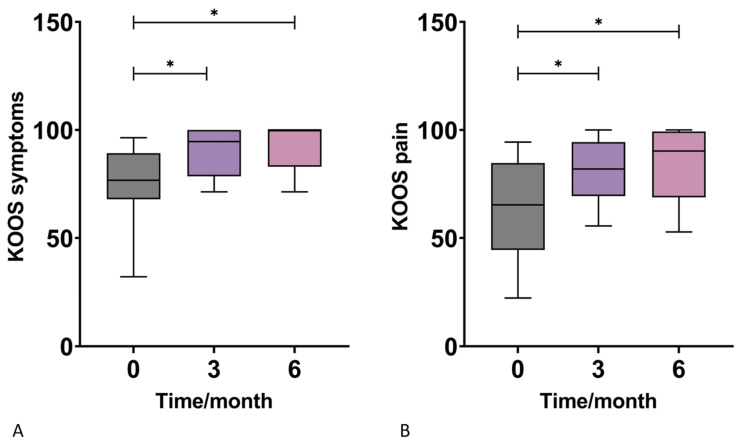

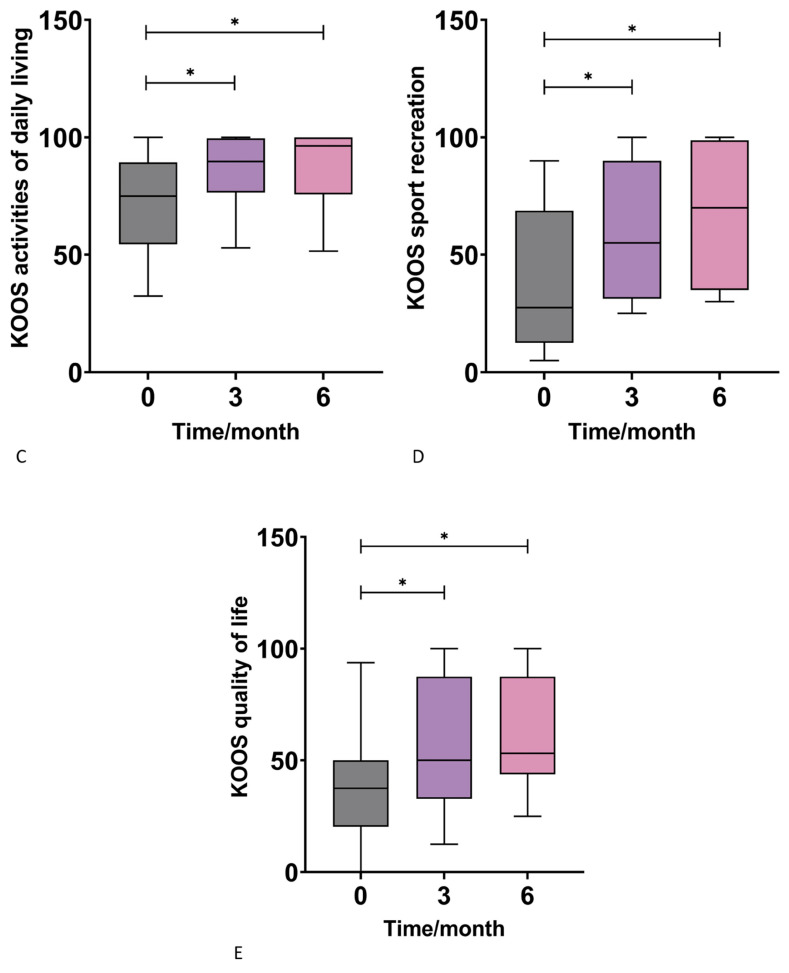
Box-plots show the distribution of Knee Injury and Osteoarthritis Outcome Scores (KOOS) subscores for symptoms (**A**), pain (**B**), activities of daily living (**C**), sport and recreation function (**D**), and quality of life (**E**) at baseline and at 3 and 6 months after application of autologous conditioned adipose tissue and leukocyte-poor platelet-rich plasma. There was a significant improvement in all subscores at 3 and 6 months after the therapeutic intervention when compared to the baseline. *—*p* < 0.05 (Wilcoxon test).

There were statistically significant increases in the KOOS symptoms subscores across all time points (three and six months) when compared to the baseline score before intervention. The median value steeply increased from the baseline of 76.79 to 94.64 at three months and finally reached 100.00 at the end of the six month follow-up (Figure 2A, Table 2). Furthermore, a statistically significant increase was observed in the KOOS pain scores at three and six months when compared to the baseline score before intervention. A steady increase from the median baseline value of 65.28 to 81.94 at three months and then 90.28 was noted (Figure 2B, Table 2). A statistically significant increases in the KOOS Activities of Daily Living subscore were observed after the therapeutic intervention when compared to the baseline scores. The mean scores increased from 75.00 to 89.71 at three months and then finally to 96.32 at six months (Figure 2C, Table 2). The KOOS Sport and Recreation Function subscore was also found to be statistically significant by the increase in values when compared to the baseline scores (Figure 2D, Table 2). Median values rose from 27.50 to 55.00 at the three months follow-up and at six months it increased further to 70.00. Increases in KOOS Quality of Life subscores values were found (Figure 2E, Table 2). A statistically significant improvement in the KOOS Quality of life score was observed by a rise in median values from the baseline 37.50 to 50.00 at three months and 53.13 at the six months follow-up.

### 3.3. Western Ontario and McMaster Universities Osteoarthritis Index (WOMAC)

**Figure 3:**
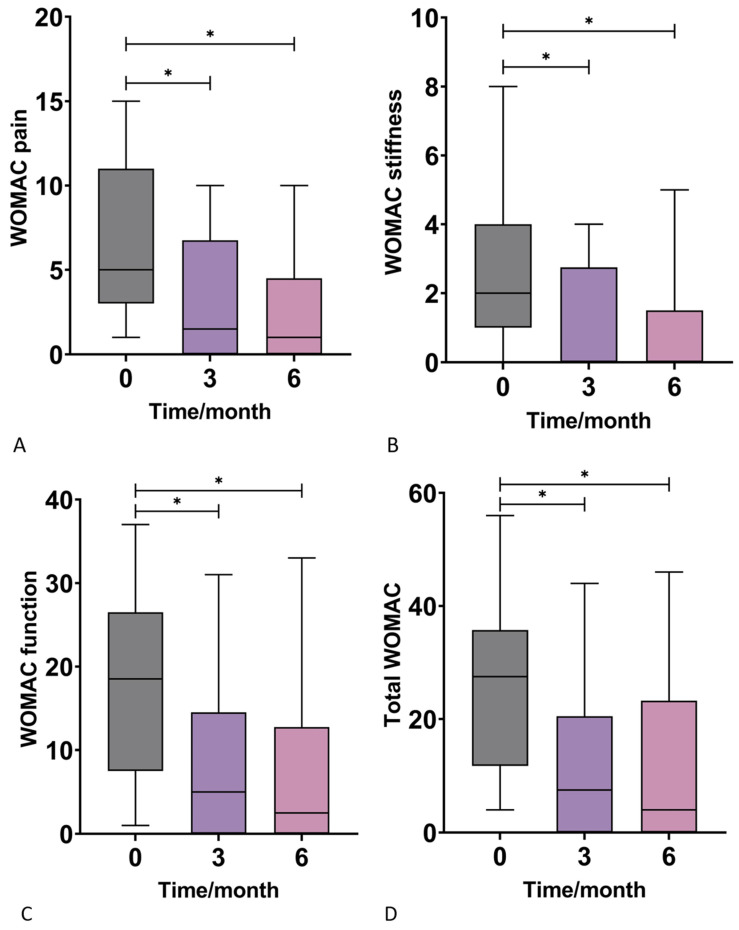
Box-plots show the distribution of Western Ontario and McMaster Universities Osteoarthritis Index (WOMAC) subscores for pain (**A**), stiffness (**B**), function (**C**), and the total score (**D**) at baseline and at 3 and 6 months after application of autologous conditioned adipose tissue and leukocyte-poor platelet-rich plasma. There was a significant improvement in all subscores 3 and 6 months after the therapeutic intervention compared to the baseline. *—*p* < 0.05 (Wilcoxon test).

There was a statistically significant decrease in the WOMAC pain subscores across all time points (three and six months) when compared to the baseline score before intervention. The median value steeply decreased from the baseline of 5.00 to 1.50 at three months and reached 1.00 at the end of the 6-month follow-up (Figure 3A, Table 2). Furthermore, a statistically significant decrease was observed in the WOMAC Stiffness scores at three and six months when compared to the baseline score. A decrease from the median baseline value of 2.00 to 0.00 at three and six months was noted (Figure 3B, Table 2). A statistically significant decrease in the WOMAC function subscore was observed after the therapeutic intervention when compared to the baseline scores. The mean scores decreased from 18.50 to 5.00 at three months and then to 2.50 at six months (Figure 3C, Table 2). Finally, the total WOMAC score significantly decreased from the median baseline value of 27.50 to 7.50 three months after the intervention and to 4.00 at the six month follow-up (Figure 3D, Table 2).

### 3.4. dGEMRIC

**Figure 4:**
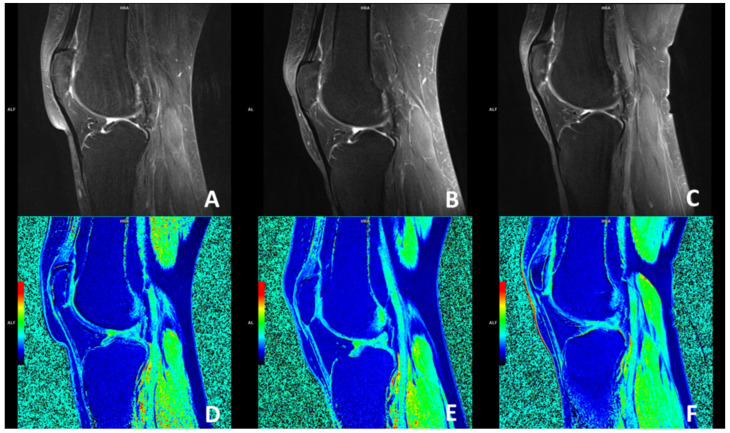
Sagittal MRI magnetic resonance imaging (MRI) slices through the center of the knee accessing the patellofemoral joint osteoarthritic changes using fat-suppressed proton-density-weighted turbo spin-echo method at baseline (**A**), 3 months (**B**), and 6 months (**C**) follow-ups with corresponding delayed gadolinium-enhanced MRI of the cartilage (dGEMRIC) images (**D**–**F**). No changes were seen when dGEMRIC indices were calculated.

The magnetic resonance imaging using the dGEMRIC index showed no significant improvement in the glycosaminoglycan (GAG) composition of the cartilage in the knees treated with ACA-SVF and LP-PRP at 3- and 6-month follow-ups when compared to the baseline values for each of the analyzed compartments, including medial and lateral femur, medial and lateral tibia, trochlea, and medial and lateral patella (Figure 4).

## 4. Discussion

The results of the presented study indicate that patients experienced statistically significant clinical improvements, as seen in the reduction in the VAS and WOMAC scores and the increase in KOOS from the baseline values to the follow-up periods of three and six months. These changes were observed in the total test scores and in the test subcategories. This is in line with previous studies [23,24,25,26,27]. It was shown earlier that SVF and PRP can influence paracrine activity through the various factors secreted, which include: PDGF, TGF, VEGF, EGF, FGF, CTGF, IGF-1, HGF, KGF, Ang-1, PF4, SDF-1, and TNF [17,28,29,30]. It is the presence of these factors that are responsible for the anti-inflammatory effects and are the likely contributors to the improved scores at three and six months [17,28,29,30]. According to Baria et al. a patient’s degree of activity before the treatment could influence the clinical outcomes because it could influence the PRP content [31]. In our research, the physical activity of patients was not reported before the study. Previous research with MFAT has shown a statistical improvement in the dGEMRIC values indirectly showing the glycosaminoglycan (GAG) concentration in the hyaline cartilage [9,10]. However, this was not observed in the present study. A possible hypothesis explaining this observation might lay in the quantitative presence of MSCs in MFAT when compared to that of ACA. While the work by Zenić et al. proved that the same population of cells (after treating the samples with 1% collagenase type I) is present in identical ratios in both MFAT and ACA, the results in this study might be explained by the ACA method used in this study as an inefficient methodology in obtaining a significant amount of SVF or by the insufficient cell quantity. [32]. In addition, the total cellular count obtained by the ACA method used in this study (2 mL) may be less in comparison with the larger volume samples of MFAT from other systems. Available SVF, as well as a larger quantity of cells could directly influence cartilage regeneration However, there is no consensus on the dosage of MSCs [33]. Low and high dosages have both been proven to have a beneficial effect [34]. Gupta et al. showed that the optimal dose of BM-MSCs might be around 25 × 10^6^ cells, while higher doses were associated with higher adverse events [35]. Other studies have found that doses between 2 × 10^6^ and 60 × 10^6^ of MSCs, when applied at a greater frequency, were potentially within a therapeutic range [36]. Eventually, the paracrine effect of MSCs derived from adipose tissue plays a critical role in cartilage regeneration. Additionally, there is no doubt that the MFAT produced by different systems is likely to differ in cellular content, which can directly affect the paracrine effect (cytokine secretion) of mesenchymal stem cells.

Furthermore, Chalal et al. found that higher doses of 50 × 10^6^ BM-MSCs resulted in a lessening of synovitis and an improvement in the WOMAC scores [37]. Another possible hypothesis is that the perivascular milieu is less disrupted in MFAT than in ACA-SVF. As evidenced, pericytes are an in vivo origin of MSCs and play a role in the chondrogenic potentials of MSCs [38,39]. Therefore, a less disrupted milieu due to minimal manipulation, or centrifugation, might result in a better function of this important cell group and thus result in better a chondrogenic potential in vivo [40,41]. However, collagenase-derived SVF preparations were shown to have a greater chondrogenic potential in vitro than their mechanically derived counterparts [11,42]. Accordingly, a direct comparison of different SVF results might lead to false conclusions as the method of extraction in the majority of research is not clearly identified. However, similar results to MFAT were seen when comparing studies using enzymatically prepared SVF [12,13,14,15]. As such, the clinical effects of ACA-SVF with LP-PRP therapy could be the result of the anti-inflammatory effects from the cells and growth factors present [42,43]. Further building on these findings, several studies have concluded that a more standardized approach should be conducted in terms of which PRP formulation should be used [43], intending to answer questions posed by currently available guidelines and with the goal of an eventual inclusion in the guidelines [17,33].

The limitations of this study include a small patient size (the reason for which the study did not consider age and BMI in the interpretation of results). A further limitation is the addition to the LP-PRP with PPP to fulfill the volume deficit in the final end product and an undetermined amount of MSCs that were delivered intraarticularly. The latter is a constant limiting factor in all the studies in this field, which should be defined in further work.

## 5. Conclusions

A combination therapy of ACA and LP-PRP provides excellent clinical and statistically significant improvements in symptoms in patients with mild to moderate KOA. Overall, the growing body of evidence supports SVF with PRP as a minimally invasive approach in the management of KOA. However, the cellular composition of MFAT plays a critical role in cartilage regeneration. Mesenchymal stem cells from adipose tissue provide an excellent safety profile and favorable outcomes for patients based on observed pain and joint function. However, there needs to be a more structured experimental approach, along with standardization of the terminology concerning the application of all forms of MSCs and the objectification of outcomes. Furthermore, a more structured selection of patients regarding KOA staging is necessary before making a final conclusion.

## Figures and Tables

**Table 1:** Patient inclusion and exclusion criteria.

Patient inclusion criteria	patients with KOA patients older than 18 years and younger than 75 years
Patient exclusion criteria	patients with malignant disease patients with systemic inflammatory diseases (e.g., rheumatoid arthritis) patients with diffuse grade IV chondromalacia according to the ICRS classification patients with an unstable knee on clinical exam or with visible anterior cruciate ligament tear on MRI patients with acute meniscal lesions or injuries of other knee structures as the main cause of pain and other symptoms patients with a history of knee surgery patients with mental illness (patients in whom cooperation cannot be expected during the study) patients who are found to be unable to respond to follow-up examinations

**Table 2:** Median values of VAS, KOOS, and WOMAC scores. M—median; IQR—interquartile range; SD—standard deviation; T0—baseline score; T3—score after 3 months; T6—score after 6 months; VAS—Visual analog scale; KOOS—Knee Injury and Osteoarthritis Outcome Score; WOMAC—Western Ontario and McMaster Universities Osteoarthritis Index.

		T0	T3	T6	*p* Value (Kruskal-Wallis Test)
VAS-Rest	M	3.00	1.00	0.50	<0.001
	IQR	2.00	2.00	2.00	
	SD	±1.797	±1.063	±1.628	
VAS-Active	M	6.00	2.00	2.00	0.002
	IQR	3.00	4.75	5.00	
	SD	±2.066	±2.613	±2.277	
KOOS Symptoms	M	76.79	94.64	100.00	0.001
	IQR	21.43	21.43	16.96	
	SD	±16.24	±10.93	±9.873	
KOOS Pain	M	65.28	81.94	90.28	0.017
	IQR	40.28	25.00	30.56	
	SD	±21.42	±15.00	±17.22	
KOOS Activities of Daily Living	M	75.00	89.71	96.32	0.014
	IQR	34.93	23.16	24.26	
	SD	±20.00	±14.91	±15.37	
KOOS Sport and Recreation Function	M	27.50	55.00	70.00	0.006
	IQR	56.25	58.75	63.75	
	SD	±28.72	±29.71	±29.42	
KOOS Quality of Life	M	37.50	50.00	53.13	0.064
	IQR	29.69	54.69	43.75	
	SD	±26.81	±29.75	±27.28	
WOMAC Pain	M	5.00	1.50	1.00	0.009
	IQR	8.00	6.75	4.50	
	SD	±4.423	±3.637	±3.856	
WOMAC Stiffness	M	2.00	0.00	0.00	0.014
	IQR	3.00	2.75	1.50	
	SD	±2.160	±1.455	±1.559	
WOMAC Function	M	18.50	5.00	2.50	0.006
	IQR	19.00	14.50	12.75	
	SD	±10.92	±9.793	±9.858	
Total WOMAC	M	27.50	7.50	4.00	0.005
	IQR	24.00	20.50	23.25	
	SD	±15.75	±13.87	±14.08	
		T0	T3	T6	*p* value (Friedman test)
VAS-Rest	M	3	1	0.5	<0.0001
	IQR	2	2	2	
	SD	±1.797	±1.063	±1.628	
VAS-Active	M	6	2	2	<0.0001
	IQR	3	4.75	5	
	SD	±2.066	±2.613	±2.277	
KOOS Symptoms	M	76.79	94.64	100	<0.0001
	IQR	21.43	21.43	16.96	
	SD	±16.24	±10.93	±9.873	
KOOS Pain	M	65.28	81.94	90.28	<0.0001
	IQR	40.28	25	30.56	
	SD	±21.42	±15.00	±17.22	
KOOS Activities of Daily Living	M	75	89.71	96.32	0.0001
	IQR	34.93	23.16	24.26	
	SD	±20.00	±14.91	±15.37	
KOOS Sport and Recreation Function	M	27.5	55	70	<0.0001
	IQR	56.25	58.75	63.75	
	SD	±28.72	±29.71	±29.42	
KOOS Quality of Life	M	37.5	50	53.13	0.0004
	IQR	29.69	54.69	43.75	
	SD	±26.81	±29.75	±27.28	
WOMAC Pain	M	5	1.5	1	<0.0001
	IQR	8	6.75	4.5	
	SD	±4.423	±3.637	±3.856	
WOMAC Stiffness	M	2	0	0	<0.0001
	IQR	3	2.75	1.5	
	SD	±2.160	±1.455	±1.559	
WOMAC Function	M	18.5	5	2.5	0.0003
	IQR	19	14.5	12.75	
	SD	±10.92	±9.793	±9.858	
Total WOMAC	M	27.5	7.5	4	0.0002
	IQR	24	20.5	23.25	
	SD	±15.75	±13.87	±14.08	

## Data Availability

The data presented in this study are available on request from the corresponding author.
